# Exploring the Diversity and Distribution of Medico-Veterinary Fungal Diseases in Africa: Harnessing a Multisectoral One Health Strategy for Cost-Effective Prevention and Preparedness

**DOI:** 10.3390/jof11080569

**Published:** 2025-07-30

**Authors:** Ayman Ahmed, Nouh Saad Mohamed, Emmanuel Edwar Siddig

**Affiliations:** 1 Pan-Africa One Health Institute (PAOHI), Kigali 11KG St203, Rwanda; nouh_saad@outlook.com; 2 Institute of Endemic Diseases, University of Khartoum, Khartoum, Sudan; 3 Swiss Tropical and Public Health Institute (Swiss TPH), Allschwil, Switzerland; 4 Faculty of Sciences, University of Basel, Petersplatz 1, Basel, Switzerland; 5 Faculty of Medical Laboratory Sciences, University of Khartoum, Khartoum 11111, Sudan; 6 Pan Africa Biomedical Institute, Kigali, Rwanda

**Keywords:** zoonotic fungal diseases, Africa, public health, One Health approach, surveillance, diagnostic capacity

## Abstract

The diversity and distribution of medical and veterinary-relevant fungal diseases in Africa underscore the critical need for a multisectoral One Health strategy to enhance cost-effective preparedness and prevention. This review explores the geographic spread and epidemiology of key medical and veterinary fungi, including *Emergomyces*, *Blastomyces*, *Coccidioides*, *Cryptococcus*, *Dermatophytes*, *Histoplasma*, *Sporothrix*, *Talaromyces*, *Paracoccidioides*, *Aspergillus*, and *Malassezia*. Evidence indicates that many of these infections remain underdiagnosed and underreported, especially in vulnerable immunocompromised populations, due to limited surveillance, diagnostic capacity, and awareness. The increasing prevalence of these diseases, often in tandem with rising HIV rates and environmental changes, highlights the urgent need for coordinated efforts across human, animal, and environmental health sectors. Implementing comprehensive, multisectoral interventions—focused on enhancing diagnostic capabilities, public awareness, surveillance, and cross-sector collaboration—is vital for effective prevention and control of these emerging fungal threats in Africa.

## 1. Introduction

Fungal infections represent a significant and often overlooked public health challenge globally [1,2,3]. With approximately one billion individuals affected by fungal diseases each year, resulting in an estimated 1.5 million deaths, the impact of these infections is profound [4]. Over the past century, there has been a marked increase in both the prevalence of fungal infections and associated fatalities [5]. This rise has been particularly pronounced among immunocompromised individuals, leading to the emergence of several new and previously known fungal infections [6]. Notably, a subset of these infections is classified as zoonotic, meaning they can be transmitted from animals to humans [7].

Zoonotic diseases, traditionally viewed as predominantly viral or bacterial in nature, encompass a significant number of fungal infections that have drawn less attention in public health discussions [8,9]. Animals have been identified as key reservoirs for many infectious diseases, accounting for approximately 75% of all emerging infections. The transmission of fungal pathogens from animals to humans is a growing concern, particularly in regions like Africa, where close human–animal interactions are prevalent [10,11]. This review examines the distribution of various fungi with medical and veterinary relevance (Table 1), some of which have zoonotic potential, relevant to the African continent (Appendix A), examining their geographical distribution and the public health implications associated with these infections.

## 2. Medically Important Fungi with the Potential for Zoonotic Transmission to Humans in Africa

### 2.1. Emergomycosis

**Figure 1:**
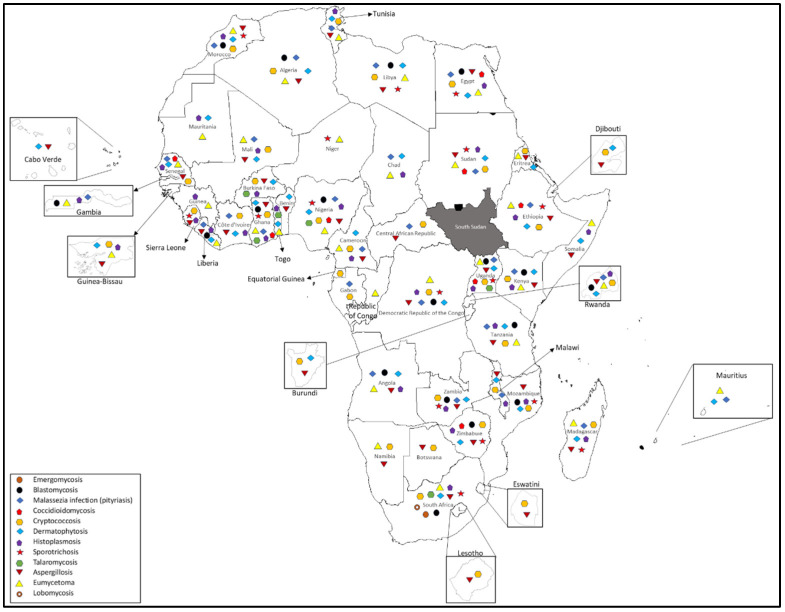
Showing the distributions and diversity of medically important zoonotic fungal diseases among different African countries.

Emergomycosis is a serious fungal infection caused by a group of fungi known as *Emergomyces*, which was previously referred to as *Emmonsia* [12]. There are five known species of this fungus, including *Es. pasteurianus*, *Es. africanus*, *Es. canadensis*, *Es. orientalis*, and *Es. europaeus*. Emergomyces can be found worldwide, but certain species, like *Es. pasteurianus* and *Es. africanus*, are predominantly found in Africa, particularly in South Africa, where the highest number of cases has been recorded [13]. *Es. africanus* is a recently identified species that appears to only exist in southern Africa [14]. This fungus is thermally dimorphic, meaning it can change form depending on temperature. It primarily affects individuals with advanced HIV, and infections occur when fungal spores are inhaled from the soil [12]. Cases of emergomycosis have been documented on four continents: Asia, Europe, Africa, and North America [15]. Given the increase in global HIV cases, it is likely that emergomycosis exists in many areas without being reported, leading to underdiagnosis. To date, all reported cases have come from South Africa (Figure 1), with no other African countries reporting infections.

### 2.2. Blastomycosis

Blastomycosis is a fungal infection caused by species of the dimorphic fungus Blastomyces, which belongs to the family Ajellomycetaceae and the genus Blastomyces. It occurs when spores from the Blastomyces species—commonly found in soil—are inhaled. The species responsible for blastomycosis include *B. dermatitidis*, *B. percursus*, *B. helices*, *B. emzantsi*, *B. parvus*, *B. silverae*, and *B. gilchristi* [16].

In Africa, the primary causative agent is *Blastomyces percursus* [17]. The infection typically presents as pulmonary disease, which can progress to skin lesions, and it may also affect other organs such as the brain. Remarkably, extra-pulmonary manifestations are the most common form of blastomycosis. Pulmonary involvement, when assessed through imaging, shows signs such as alveolar infiltrates, consolidation, and cavitation on chest radiographs [18,19]. Reported cases of blastomycosis have emerged from several African countries, including Kenya, Mozambique, Rwanda, Tanzania, Uganda, Zambia, Zimbabwe, The Gambia, Ghana, Liberia, Nigeria, Angola, the Democratic Republic of the Congo, South Africa, Algeria, Egypt, the Libyan Arab Jamahiriya, Morocco, and Tunisia (Figure 1). Awareness and monitoring of this infection are crucial, particularly in areas where exposure to contaminated soil is prevalent.

### 2.3. Coccidomycosis

Coccidioidomycosis, commonly known as Valley Fever, is caused by inhaling spores from the *Coccidioides* fungi [20]. *C. immitis* and *C. posadasii* are two significant species associated with this disease [21]. *C. posadasii* thrives in arid areas of the southwestern United States, northern Mexico, and South America, while *C. immitis* is primarily found in California’s San Joaquin Valley [21]. Coccidioidomycosis transmission predominantly occurs during the dry summer and late fall months, when soil disturbances such as wind and storms are frequent. Rarely, infection can also result from contact with contaminated materials [22]. Its prevalence is extremely low outside the Americas, but cases have been documented among travelers returning from endemic regions in Asia and Europe [22]. In Africa, Coccidioidomycosis has been reported in Ethiopia, Sudan, Uganda, Zimbabwe, Ghana, Nigeria, Egypt, and Senegal (Figure 1). Public health initiatives should focus on improving awareness and education about this disease, especially for individuals traveling to endemic areas and for those living in regions where the *Coccidioides* fungi are present.

### 2.4. Cryptococosis

*Cryptococcus* species are ubiquitous, encapsulated yeasts found worldwide and are commonly associated with environmental exposures, including pigeon droppings, soil, water, and certain foods. Cryptococcosis has emerged as one of the most lethal fungal diseases globally, with the annual death toll exceeding 180,000 [23,24]. The classification of *Cryptococcus* species has undergone changes based on molecular studies, leading to a more refined understanding of their genetic diversity. *Cryptococcus neoformans* is now divided into two species: *Cryptococcus neoformans sensu stricto* (formerly *Cryptococcus neoformans var. grubii*) and *Cryptococcus deneoformans* (formerly *Cryptococcus neoformans* var. neoformans). Similarly, *Cryptococcus gattii* is now recognized as a complex of five species: *Cryptococcus gattii sensu stricto*, *Cryptococcus bacillisporus*, *Cryptococcus deuterogattii*, *Cryptococcus tetragattii*, and *Cryptococcus decagattii*.

This disease is particularly dangerous, as it affects both immunocompromised and immunocompetent individuals, often by causing meningoencephalitis in HIV-positive patients. The primary causative agent is *Cryptococcus neoformans*, while *Cryptococcus gattii* has been increasingly observed in healthy individuals, leading to pulmonary infections. Since its first case was reported in the mid-1980s, the incidence of cryptococcosis has risen, underscoring the necessity for heightened public health measures [23]. In Africa, the disease has been reported in multiple countries, including Burundi; Djibouti; Eritrea; Ethiopia; Kenya; Madagascar; Malawi; Mozambique; Rwanda; Sudan; Tanzania; Uganda; Zambia; Zimbabwe; Burkina Faso; Cameroon; Côte d’Ivoire; Ghana; Guinea-Bissau; Mali; Nigeria; Senegal; Sierra Leone; Central African Republic; the Democratic Republic of the Congo; Equatorial Guinea; Gabon; Lesotho; Swaziland; Botswana; Namibia; South Africa; Algeria; Egypt; Libyan Arab Jamahiriya; Morocco; and Tunisia (Figure 1).

### 2.5. Dermatophytosis

Dermatophytosis, commonly referred to as ringworm or tinea, is a prevalent fungal infection observed globally [25]. However, recent trends indicate significant changes in the epidemiology of this disease [25]. The types of fungal species causing dermatophytosis now vary greatly across different countries. This shift has been associated with factors such as inappropriate treatment practices, high population density, international travel, and migration [25,26]. Additionally, the increasing popularity of pets has led to the emergence of new species, such as *Trichophyton erinacei* and *Trichophyton benhamiae*, which are contributing to a rise in zoonotic infections [27,28]. The rising incidence of dermatophytosis raises serious public health concerns, as the costs associated with treatment can be substantial and prolonged. Such factors often lead to non-adherence to prescribed therapies, resulting in potential resistance to antifungal medications [25,29]. A particularly alarming situation has emerged with a large-scale outbreak of dermatophytosis currently ongoing in India, which is beginning to spread to other regions, including parts of Africa [30,31]. This outbreak is characterized by human-to-human transmission, typical of dermatophytes that primarily infect humans, yet it exhibits a severity of symptoms usually associated with zoophilic dermatophytes [32]. The outbreak has been linked to a novel species known as *Trichophyton indotineae*, which demonstrates enhanced virulence compared to the previously widespread species *Trichophyton mentagrophytes* and *Trichophyton interdigitale* [31,33].

Research suggests that *T. mentagrophytes*, which typically infects wild animals, is adapting, moving to domestic animals, and behaving as an anthropophilic clonal variant. This Indian strain has shown a concerning trend of increased antifungal resistance, likely due to the overuse of common antifungal agents by the public, complicating control efforts and leading to a rising incidence of the disease [7].

In Africa, dermatophytosis has been reported in numerous countries, highlighting the need for greater awareness and intervention. The countries affected include Burundi, Djibouti, Eritrea, Ethiopia, Kenya, Madagascar, Malawi, Mauritius, Mayotte, Mozambique, Réunion, Rwanda, Somalia, Sudan, Tanzania, Uganda, Zambia, Benin, Burkina Faso, Cameroon, Cabo Verde, Côte d’Ivoire, Ghana, Guinea-Bissau, Liberia, Mali, Mauritania, Nigeria, Senegal, Togo, Angola, Chad, the Democratic Republic of the Congo, South Africa, Algeria, Egypt, the Libyan Arab Jamahiriya, Morocco, and Tunisia (Figure 1).

### 2.6. Histoplasmosis

Histoplasmosis is a significant fungal infection primarily found in the Ohio and Mississippi river valleys of the United States, as well as in various regions across Central and South America, Western, Southern, Eastern, and Central Africa, and Southeast Asia [34]. The infection is caused by two distinct varieties of the fungus *Histoplasma capsulatum*: *Histoplasma capsulatum var. capsulatum* (Hcc), which is the classical form of the disease, and *Histoplasma capsulatum var. duboisii* (Hcd), which is associated with the African form of histoplasmosis [35]. Transmission of histoplasmosis predominantly occurs through the inhalation of microconidia, the airborne spores of the fungus [35]. A key risk factor for developing severe histoplasmosis is HIV/AIDS, which has led to the classification of the disease as an AIDS-defining illness since 1987. Globally, it is estimated that around 500,000 individuals are diagnosed with histoplasmosis each year. Of these cases, approximately 100,000 progress to disseminated histoplasmosis, and around 25,000 individuals succumb to the infection [36]. The disease is increasingly being recognized in various parts of the world. While histoplasmosis was initially endemic to regions such as the Ohio and Mississippi river valleys and sub-Saharan Africa, its reach has expanded to the Caribbean, Southeast Asia, and throughout South and Central America.

The prevalence of histoplasmosis may be underestimated due to frequent misdiagnosis, as it is often confused with conditions such as tuberculosis or emergomycosis. Moreover, in many countries, the requirement for reporting histoplasmosis infections to health authorities is not mandatory, hindering accurate tracking of the disease’s distribution and prevalence. Histoplasmosis has been reported in numerous countries across Africa, including Ethiopia, Kenya, Rwanda, Tanzania, Uganda, and Somalia; South Africa, Zimbabwe, Zambia, Mozambique, Malawi, and Angola; Burkina Faso, Cameroon, Côte d’Ivoire, The Gambia, Ghana, Guinea, Guinea-Bissau, Liberia, Mali, Mauritania, Nigeria, Senegal, Sierra Leone, and Togo; and Egypt, Morocco, and Tunisia (Figure 1).

### 2.7. Sporotrichosis

Sporotrichosis is a subacute to chronic fungal infection caused by thermally dimorphic fungi classified within the Ascomycota division. These fungi belong to the order Ophiostomatales, the family Ophiostomataceae, and the genus *Sporothrix* [37]. Commonly referred to as “Rose Gardener’s Disease,” sporotrichosis primarily results from infection with the saprophytic, thermally dimorphic fungus *Sporothrix schenckii* [37]. Traditionally, *Sporothrix schenckii* was the sole species identified within this genus; however, recent phenetic and molecular genetic studies have revealed the existence of additional species within the genus [37]. These advances have expanded our understanding of the diversity and epidemiology of sporotrichosis, highlighting its significance as a fungal pathogen capable of causing both localized and systemic infections in humans and animals.

This disease is predominantly found in tropical and subtropical regions and is typically transmitted through a traumatic injury that allows fungal spores to enter the host, classifying it as an implantation mycosis [37].

The clinical manifestations of sporotrichosis can be broadly categorized into skin, mucosal, systemic, and immunoreactive forms. The most common presentations include cutaneous or subcutaneous lesions and lymphadenopathy, particularly in cases of disseminated disease [38,39]. Although sporotrichosis is rarely life-threatening, it can lead to significant morbidity and a marked decline in quality of life. The ecological niche of *Sporothrix* fungi is primarily found in soil and decaying plant matter [37]. Infection often occurs during activities such as farming, gardening, animal husbandry, and mining, where there is increased risk of exposure to the spores. Zoonotic transmission is also prevalent, with cases frequently linked to interactions with infected animals.

Recently, sporotrichosis has been recognized as one of the deep mycoses classified among neglected tropical diseases. The ecology and epidemiology of this infection exhibit variations across different geographical regions, contributing to its distinct patterns of occurrence. Reported cases of sporotrichosis have emerged from various countries, including Ethiopia, Madagascar, Mozambique, Sudan, Uganda, Zambia, Zimbabwe, Ghana, Niger, Nigeria, Sierra Leone, the Democratic Republic of the Congo, South Africa, Egypt, Libya, and Morocco (Figure 1).

### 2.8. Talaromycosis

Talaromycosis, formerly referred to as penicilliosis, is a fungal infection stemming from the organism *Talaromyces marneffei* (previously known as *Penicillium marneffei*) [40]. This disease is primarily found in East and Southeast Asia and is classified as a neglected tropical illness [40].

Patients may exhibit a variety of symptoms, including non-painful skin lesions, particularly on the face and neck, fever, anemia, swollen lymph nodes, and hepatic involvement. It predominantly affects individuals with weakened immune systems, such as those living with HIV/AIDS, cancer patients, individuals who have undergone organ transplants, those on long-term corticosteroid therapy, the elderly, malnourished persons, or individuals with autoimmune disorders [41].

*Talaromyces marneffei* is notable for being the only thermally dimorphic fungus within the *Talaromyces* genus [41,42]. This means that, like many other dimorphic fungi, it exists as a mold in external environments but transforms into small, round yeast cells when it infects a host’s tissues. Although there is limited information available regarding its natural habitat, the fungus has been retrieved from soil samples [41,42]. Research suggests that heavy rainfall may create conditions conducive to the growth and spread of this fungus. The transmission of the infection is believed to occur through the inhalation of fungal spores that originate from unidentified sources in the environment. The incubation period for *Talaromyces marneffei* can differ, and it is possible for the infection to remain dormant and asymptomatic for extended periods [41,42]. Cases have also been identified in various regions across Africa, including Uganda, Burkina Faso, Ghana, Nigeria, Togo, and South Africa (Figure 1).

### 2.9. Lobomycosis

Lobomycosis is a rare cutaneous fungal infection primarily caused by *Lacazia loboi*. Its distribution is almost exclusive to the Americas, and it has a particularly high prevalence in the Amazon basin [43]. Cases of lobomycosis have been reported only in dolphins and humans [43]. The fungus had been identified in the soil and vegetation, as well as in aquatic environments [43]. In Africa, it has been reported in South Africa (Figure 1). With limited cases documented, this infection emphasizes a gap in our understanding of its transmission dynamics and risk factors within the region. Further investigation is warranted to develop appropriate public health responses.

### 2.10. Paracoccidioidomycosis

Paracoccidioidomycosis is a systemic fungal infection caused by two thermally dimorphic fungi: *Paracoccidioides brasiliensis* and *Paracoccidioides lutzii*. It primarily occurs in the humid subtropical regions of Latin America, including Brazil, Argentina, Colombia, and Venezuela, as well as parts of Central America [44,45,46,47,48]. The natural habitat of the fungus is still undetermined; however, it is hypothesized that the fungus is able to survive and proliferate in the soil [49]. The fungus then enters via the respiratory tract or from injuries through the skin and mucous membranes [49].

The lungs are the main site of infection, which typically results from inhaling conidia and mycelial fragments. In many cases, the infection is asymptomatic. There are two main forms of paracoccidioidomycosis: the acute/subacute form, often referred to as juvenile paracoccidioidomycosis, and the chronic form, also known as adult paracoccidioidomycosis [50]. The acute or subacute forms account for approximately 10% of clinical cases and are typically seen in children and adolescents under 16 years old, affecting both genders equally. Common clinical features include lymphadenopathy, hepatosplenomegaly, fever, weight loss, malaise, and various skin lesions, while respiratory symptoms and mucous membrane involvement are rare [51]. In contrast, the chronic form is more common in adults over 16, with a notable male-to-female ratio of 20:1, likely due to the inhibitory effects of estrogen on the conversion of mycelial forms to yeast. Patients may exhibit primary lung infections, cough, dyspnea, fever, weight loss, and complications related to chronic lung disease, including fibrosis and emphysema. Additional symptoms may involve mucous membrane lesions, skin lesions, and cervical lymphadenopathy. Risk factors for developing paracoccidioidomycosis include agricultural occupations, malnutrition, smoking, and alcohol use [52,53].

Notably, paracoccidioidomycosis has not been documented in Africa, raising questions about whether the disease is genuinely absent from the continent or whether underreporting and inadequate surveillance have led to missed cases.

### 2.11. Aspergillosis

*Aspergillus* is a ubiquitous filamentous fungus belonging to the genus *Aspergillus* in the family Trichocomaceae [54,55,56]. It primarily causes infections in immunocompromised individuals and those with underlying pulmonary conditions [57,58,59,60,61,62]. In the environment, *Aspergillus* species obtain nutrients from dead organic material and reproduce asexually through conidia. Over 24 species of *Aspergillus* are capable of causing human disease, with *A. fumigatus* being the most commonly implicated pathogen, followed by *A. terreus and A. flavus* [54,63]. Although caused by fungi within the same genus, aspergillosis encompasses a broad spectrum of clinical conditions that vary widely depending on the host’s immune status. This infection can range from allergic reactions to severe invasive respiratory diseases. *Aspergillus* spores are widespread in soil, plant debris, and decaying organic matter, and most individuals inhale these spores without experiencing health issues. However, those with weakened immune systems, pre-existing lung diseases, or other risk factors may develop a variety of health problems stemming from exposure to these fungi [63].

*Aspergillus* species are distributed widely across the globe, thriving in both indoor and outdoor environments. They are especially prominent in tropical and subtropical regions, where humidity and temperature favor their growth. They are present in many countries across Africa, including Burundi; Djibouti; Eritrea; Ethiopia; Kenya; Madagascar; Malawi; Mozambique; Rwanda; Somalia; Sudan; Tanzania; Uganda; Zambia; Zimbabwe; Benin; Burkina Faso; Cameroon; Cabo Verde; Côte d’Ivoire; Ghana; Guinea-Bissau; Liberia; Mali; Nigeria; Senegal; Sierra Leone; Angola; Central African Republic; the Democratic Republic of the Congo; Lesotho; Swaziland; Botswana; Namibia; South Africa; Algeria; Egypt; Libyan Arab Jamahiriya; Morocco; and Tunisia (Figure 1).

### 2.12. Eumycetoma

Eumycetoma is a chronic, granulomatous infectious disease primarily characterized by a triad of clinical features: painless swelling of the affected tissue, the formation of discharging sinuses, and the extrusion of “grains”—compact aggregations of causative microorganisms encased within a protective matrix [64]. These grains are often discharged through sinus tracts on the skin surface and serve as a hallmark diagnostic feature of the disease [64]. The condition involves a diverse and complex array of pathogenic fungi, with over 60 species implicated worldwide, making diagnosis and management particularly challenging [64]. Its global health impact is substantial, especially in endemic regions such as parts of Africa, Latin America, and Asia, where environmental and socioeconomic factors contribute to its prevalence [64,65].

In sub-Saharan Africa, the causative agents are predominantly fungal species belonging to specific genera. Most notably, fungi from the genus *Madurella* are recognized as the primary pathogens responsible for eumycetoma in these regions. Among these, *Madurella mycetomatis* is the most common, followed by *Madurella tropicana* and *Madurella fahalii* [64,65,66,67,68,69,70,71,72,73,74,75]. These fungi are considered the hallmark etiological agents and are central targets for diagnosis and treatment strategies. Beyond *Madurella*, other genera have been identified as causative agents, including *Falciformispora*, which encompasses species like *Falciformispora senegalensis* and *Falciformispora tompkinsii* [75]. These emerging pathogens complicate the diagnostic landscape due to their varied morphological and molecular profiles. Additionally, fungi such as *Aspergillus* species have been occasionally associated with eumycetoma, although less frequently, while *Chaetomium* spp., including *Chaetomium atrobrunneum*, have also been reported as causative agents in certain cases [70]. The genus *Pleurostomophora*, with *Pleurostomophora ochracea*, has been recently implicated, highlighting ongoing discoveries in the field [70].

Other less common but significant fungi include *Curvularia pseudolunata*, *Fusarium solani*, and *Microascus gracilis*. The broad diversity of causative agents reflects the complexity of eumycetoma’s etiology and underscores the necessity for precise identification via advanced diagnostic modalities such as molecular techniques [64]. Such diversity not only influences clinical management, as antifungal susceptibilities vary among species, but also emphasizes the importance of region-specific epidemiological studies to inform effective control and treatment strategies. The agents responsible for eumycetoma can infect a wide variety of hosts, including humans, animals, and plants, as well as impact agricultural and food products [64]. The disease is prevalent in diverse environmental settings worldwide, making its control and management crucial. The impact of eumycetoma extends beyond individual health, affecting animal and environmental health, and it has socio-cultural and economic factors, thereby threatening food security and safety [64].

Recent reports indicate a troubling rise in eumycetoma incidence rates, along with an increasing diversity of hosts and geographical spread. Contributing factors to this trend include climate change, globalization, poor living conditions, and inadequate hygiene and sanitation practices. These elements create an environment conducive to the spread of eumycetoma and amplify its impact on communities.

Eumycetoma has been documented in numerous countries across Africa, including Eritrea; Ethiopia; Kenya; Madagascar; Mauritius; Rwanda; Somalia; Sudan; Tanzania; Uganda; Cameroon; The Gambia; Ghana; Guinea; Guinea-Bissau; Liberia; Mali; Mauritania; Niger; Nigeria; Senegal; Togo; Angola; Chad; the Democratic Republic of the Congo; Republic of Congo; Namibia; South Africa; Algeria; Egypt; Libyan Arab Jamahiriya; Morocco; and Tunisia (Figure 1).

### 2.13. Malassezia Infection (Pityriasis)

*Malassezia* infections, commonly known as pityriasis, are conditions caused by the *Malassezia* genus of fungi, which are part of the normal skin flora in humans and other mammals [76,77]. These lipid-dependent yeasts are naturally found on the skin surface and can sometimes lead to various skin disorders, including pityriasis versicolor, a condition characterized by discolored patches on the skin [76]. *Malassezia* is ubiquitous, and its infections have been reported in various countries across the globe, indicating a widespread presence and potential for infection. The primary causative agents of pityriasis are species of the *Malassezia* genus, with *Malassezia furfur* being the most frequently implicated [78]. *Malassezia* infections are primarily associated with humans, but they can also affect other mammals, including dogs and cats [78]. In humans, the infections can occur in individuals of any age, but they are more frequently observed in adolescents and young adults, possibly due to hormonal changes affecting sebum production [78]. The transmission of *Malassezia* infections is not fully understood, as the fungi are commonly present on the skin without causing harm. Pityriasis versicolor is considered not contagious, but similar conditions may arise in susceptible hosts due to environmental triggers [78]. This infection has been reported in Ethiopia; Kenya; Madagascar; Malawi; Mauritius; Mayotte; Rwanda; Sudan; Tanzania; Uganda; Zambia; Cameroon; Côte d’Ivoire; The Gambia; Ghana; Liberia; Mali; Nigeria; Senegal; Angola; Central African Republic; Chad; the Democratic Republic of the Congo; Gabon; Algeria; Egypt; Libyan Arab Jamahiriya; Morocco; and Tunisia (Figure 1).

## 3. Conclusions and Future Perspectives

The rising incidence of zoonotic fungal diseases across Africa poses a significant challenge to public health systems, emphasizing the urgent need for a comprehensive and integrated strategy for disease prevention and management. The emergence of infections such as emergomycosis, blastomycosis, coccidioidomycosis, cryptococcosis, dermatophytosis, histoplasmosis, sporotrichosis, and eumycetoma underscores the intricate connections between human, animal, and environmental health, as encapsulated by the One Health framework. Since approximately 75% of emerging infectious diseases originate from animal reservoirs, it is vital to enhance surveillance systems and deepen our understanding of these zoonotic fungal pathogens to prevent future outbreaks and protect public health.

To effectively address this multi-dimensional public health threat, targeted interventions are necessary. Key actions include the development of robust surveillance mechanisms to monitor fungal infections in humans, animals, and the environment, ensuring the timely identification and response to emerging threats. Public health entities should collaborate closely with veterinary services and environmental agencies to establish comprehensive surveillance networks that facilitate proactive measures.

Increased awareness among healthcare professionals, veterinarians, and the public regarding zoonotic fungal infections is of paramount importance. Educational campaigns should emphasize prevention strategies, proper hygiene practices, and the critical need for early medical intervention.

Enhancing access to healthcare, particularly in rural and underserved regions, will significantly improve diagnostic capacity and treatment options. Training healthcare workers to promptly recognize signs of fungal infections can lead to timely treatment, ultimately reducing complications and fatalities. Furthermore, investing in research to understand the epidemiology, ecology, and transmission dynamics of zoonotic fungal diseases will provide essential insights to inform public health policies. Collaboration with academic institutions and international organizations will stimulate the development of innovative solutions and treatments.

Improving diagnostic capacity is crucial in addressing the burden of zoonotic fungal diseases in Africa, where there is a significant lack of diagnostic tools for fungal infections [79]. A comprehensive survey conducted in 2023 across 47 African countries assessed the diagnostic capabilities for skin fungal diseases and revealed notable disparities in availability and quality [79,80,81,82,83,84,85]. Only 15% of the countries offer skin biopsies in the public sector, while 45% do so in the private sector. However, 46% of countries perform skin biopsies regularly, mainly in university hospitals. Direct microscopy is utilized in only 42% of public sector facilities, with 21% not employing this method at all. Fungal cultures are conducted in 44% of public sector facilities, but 20% lack fungal culture services entirely, and 44% do not offer this in either the public or private sectors. Histopathological examinations are frequently used in 40% of public sector countries, but 20% do not utilize this essential diagnostic method. Economic barriers, particularly the cost of diagnostics, significantly hinder accessibility, resulting in underutilization of available services. To address these gaps, investment in advanced laboratory technologies and infrastructure is necessary, alongside the implementation of comprehensive training programs for healthcare providers in diagnostic methodologies [82,83,84,85]. Establishing specialized regional laboratories capable of conducting advanced tests and providing rapid results will support timely clinical decision-making and response to outbreaks [86]. Additionally, promoting awareness of the importance of fungal diagnostics among healthcare providers and patients, as well as implementing strategies to subsidize costs, can further improve the utilization rates of essential diagnostic services [87,88]. By enhancing the diagnostic capacity for zoonotic fungal diseases, healthcare systems in Africa can facilitate the early detection and effective management of these emerging threats, ultimately reducing their public health impact [89].

Moreover, emphasizing a One Health approach will foster collaboration across disciplines, integrating human, animal, and environmental health into one cohesive strategy. Such initiatives can help mitigate the risks associated with zoonotic diseases while enhancing overall health outcomes for communities.

In conclusion, the public health implications of emerging zoonotic fungal diseases in Africa necessitate immediate and coordinated actions. By leveraging the One Health approach, we can establish a proactive framework that recognizes the interconnectedness of health sectors, thereby improving disease prevention and control strategies. Strengthening this framework will ultimately enhance health security for individuals, animals, and ecosystems across the African continent, empowering communities to better withstand the challenges posed by these emerging pathogens.

## Figures and Tables

**Table 1:** Relevant fungal diseases and their natural history, presenting the variety of forms of infection acquisition and clinical manifestations.

Disease	Causative Agents	Animal Hosts	Mode of Transmission	Clinical Manifestation in Animals	Clinical Manifestation in Humans
Emergomycosis	*Emmonsia* spp.	Rodents	Inhalation of the fungus	Deep mycosis	Disseminated mycosis
Blastomycosis	*Blastomyces dermatitidis*	Cats, dogs, horses, marine mammals	Inhalation of airborne conidia	Cutaneous, pulmonary, disseminated infection	Cutaneous, pulmonary, disseminated infection
Coccidomycosis	*Coccidioides immitis;* *Coccidioides posadasii*	Cattle, cats, dogs, horses, snakes, reptiles, marine mammals	Inhalation of conidia and skin trauma	Self-limiting to chronic dissemination	Cutaneous, pulmonary, disseminated infection
Cryptococosis	*Cryptococcus neoformans;* *Cryptococcus gattii*	Cattle, goats, cats, dogs, horses, marine mammals	Inhalation of the fungus; implantation of the fungus into the skin	Respiratory tract, CNS, eyes, and skin	Cutaneous, eye, respiratory, and central nervous system infection
Dermatophytosis	*Microsporum* spp.; *Trichophyton* spp.	Cats, dogs, cattle, goats, horses, camels, pigs, rodents, bats	Direct contact with the infected animals or material contaminated from the site of the infection	Ring lesion with central healing and crusts at the peripheral area, some degree of folliculitis	Tinea
Histoplasmosis	*Histoplasma capsulatum*	Cattle, sheep, horses, dogs, cats, birds, bats, rats, skunks, opossums	Inhalation of the fungus	Cutaneous, pulmonary, disseminated infection	Cutaneous, pulmonary, disseminated infection
Sporotrichosis	*Sporothrix schenckii;* *Sporothix brasiliensis*	Dogs, cats, horses, cows, camels, dolphins, goats, mules, birds, pigs, rats, armadillos	Direct inoculation of the organism into skin wounds via contact with plants, soil, or penetrating foreign bodies	Lymphocutaneous, cutaneous, and disseminated	Lymphocutaneous, cutaneous, and disseminated
Talaromycosis	*Talaromyces marneffei*	Bamboo rats, dogs, cats	Unknown, but it is hypothesized that it occurs by inhalation of the fungus from the environment	Cutaneous, respiratory, and disseminated disease	Cutaneous, respiratory, and disseminated disease
Lobomycosis	*Lacazia loboi*	Dolphins	Traumatic inoculation	Cutaneous disease	Cutaneous disease
Paracoccidomycosis	*Paracoccidioides brasiliensis;* *Paracoccidioides lutzii*	Dogs, armadillos, monkeys	Inhalation of the fungus, inoculation of the organism into the subcutaneous tissues	Cutaneous (skin ulcers), adenitis, and disseminated disease	Mucocutenous, respiratory and disseminated disease
Aspergillosis	*Aspergillus* spp.	Domestic animals (dogs, horses, cats, poultry), birds, wildlife	Inhalation of airborne spores	Pulmonary mainly; cutaneous and disseminated	Pulmonary mainly; cutaneous and disseminated
Eumycetoma	More than 70 fungal species; most prevalent ones include *Madurella* spp.; *Falciformispora* spp.; *Fusarium* spp.; *Medicopsis* spp.	Cats, dogs, horses, turtles, fish, cattle, tigers	Inoculation of the causative agents into the subcutaneous tissue	Subcutaneous disease mainly; however, disseminated infection can also occur	Subcutaneous disease if the disease affected the extremities; respiratory (lung involvement); CNS
*Malassezia* infection (pityriasis)	*Malassezia* spp.	Dogs, cats, cows, sheep, pigs, horses, wild animals	Normal commensals of the skin	Dermatitis, alopecia, stenosis, otitis externa	Chronic superficial disease of the skin (pityriasis versicolor), folliculitis, seborrhoeic dermatitis and dandruff, fungaemia

## Data Availability

The original contributions presented in this study are included in the article. Further inquiries can be directed to the corresponding authors.

## Abbreviations

The following abbreviations are used in this manuscript:

AIDS	Acquired Immunodeficiency Syndrome
CNS	Central nervous system
HIV	Human Immunodeficiency Virus

## Appendix A

Data set for the diversity and distribution of zoonotic fungal diseases in Africa.
